# FTO-mediated m^6^A modification promotes malignant transformation of gastric mucosal epithelial cells in chronic Cag A^+^ *Helicobacter pylori* infection

**DOI:** 10.1007/s00432-023-04684-4

**Published:** 2023-03-15

**Authors:** Sha Cheng, Huan Li, Jingshu Chi, Wenfang Zhao, Jiahui Lin, Xiaoming Liu, Canxia Xu

**Affiliations:** Department of Gastroenterology, The Third Xiangya Hospital of Central South University, Changsha, 410013 Hunan China

**Keywords:** *Helicobacter pylori*, Cag A, FTO, m^6^A, CD44, Malignant transformation

## Abstract

**Objectives:**

Cag A^+^ *Helicobacter pylori* chronic infection cause malignant transformation of the human gastric mucosa. N6-methyladenosine (m^6^A) modifications are the most common and abundant mRNA modifications and one of the pathways affecting tumorigenicity and tumor progression. However, the role of m^6^A modification in the process of chronic *H. pylori* infection leading to malignant transformation of gastric mucosa is unclear.

**Methods:**

In this study, we used Cag A^−^ and Cag A^+^*H. pylori* chronic infection to establish cellular models in GES-1 cells and analyzed the cellular morphology, proliferation, apoptosis, invasiveness and tumorigenicity of gastric mucosal epithelial cells. The m^6^A expression levels of GES-1 cells after chronic infection with Cag A^−^ and Cag A^+^*H. pylori* were examined, and modifying effect of FTO (the fat mass and obesity-associated protein) on CD44 was verified by MeRIP–qPCR. Finally, the FTO expression changes and m^6^A expression levels were further validated in clinical gastric cancer tissues.

**Results:**

Chronic Cag A^+^*H. pylori*-infected GES-1 cells exhibit altered cell morphology, apoptosis inhibition, abnormal proliferation, enhanced migration, colony formation, and increased stem cell-like properties. Meanwhile, FTO and CD44 expression was enhanced, and FTO may induce malignant transformation of gastric mucosa by regulating CD44 mRNA m^6^A methylation modifications.

**Conclusions:**

We verified the effect of chronic stimulation of Cag A^+^*H. pylori* on malignant transformation of gastric mucosal epithelium. revealing the possibility of FTO in promoting malignant transformation of gastric mucosa by modifying CD44 mRNA methylation, suggesting that FTO expression is a potential molecule for malignant transformation of gastric mucosal epithelial cells.

**Supplementary Information:**

The online version contains supplementary material available at 10.1007/s00432-023-04684-4.

## Introduction

*Helicobacter pylori* (*H. pylori)* is one of the most contagious pathogens, infecting more than 50% of the world's population (Maixner et al. 2019). However, more than 80% of infected people have no obvious symptoms, and this highly contagious and insidious bacterium can cause life-long infection and continue to infect more people (Wang et al. 2014; Chojnacki et al. 2019; Baj et al. 2020). *H. pylori* is significantly associated with active gastritis, peptic ulcer, atrophic gastritis, intestinal metaplasia, Mail's lymphoma, and gastric cancer (Kotilea et al. 2019; Naumann and Crabtree 2004). From chronic non-atrophic gastritis, atrophic gastritis, intestinal metaplasia, dysplasia to gastric carcinogenesis, this process is called Correa's cascade (Sukri et al. 2020), usually triggered by *H. pylori* infection and influenced by synergistic genetic and environmental influences (Ansari 2018; Lahner et al. 2018). *H. pylori* is a class I carcinogen that has a significant role in malignant transformation of normal gastric mucosa into gastric carcinogenesis (Keilberg and Ottemann 2016; Maroney and Ciurli 2021). *H. pylori* can produce cytotoxin-associated gene A (Cag A), vacuolating cytotoxin A (Vac A), outer membrane proteins, urease and other virulence factors that affect host cell signaling pathways and contribute to progressive abnormal changes in the gastric epithelium (Chmiela et al. 2018). The cag pathogenicity island (cag PAI), which encodes the type IV secretion system (T4SS), injects the Cag A into host cells (Müller 2012; Merino et al. 2017), resulting in altered epithelial cell polarity, inducing the production of cells with a hummingbird phenotype, increased cell migration and cell invasion, and formation of tumor-like spheres, with a higher propensity for invasiveness and malignancy (Bessède et al. 2014; Mueller et al. 2012). It is recognized that *H. pylori* expressing cag PAI is more carcinogenic, and under chronic infection with Cag A^+^*H. pylori* the long-lasting inflammatory response and accumulation of "blows" stimulate the dysfunction and structure of gastric epithelial cells, eventually leading to the development of gastric cancer.

Gastric cancer (GC) is the fifth most common cancer and the fourth most lethal cancer in the world (Sung et al. 2021), particularly prevalent in East Asia (Patrad et al. 2022). Patients are usually first diagnosed with advanced gastric cancer with poor treatment and prognosis. Therefore, finding biomarkers and therapeutic targets for malignant transformation of the gastric mucosa is beneficial to prevent gastric cancer.

N6-methyladenosine (m^6^A) is the most abundant modification ubiquitously occurring in eukaryotic mRNAs and it plays crucial roles in tumorigenicity and tumor progression (Cai et al. 2022; Chen et al. 2019). It has been demonstrated that m^6^A methylation affects stem cell differentiation and the growth and proliferation of tumor cells. "writers" (methyltransferases, including WTAP, KIAA1429, RBM15/15B, ZC3H13 and METTL3/14/16), "readers" (YTHDF1/2/3, YTHDC1/2, IGF2BP,  HNRNPC and HNRNPA2B1) and "erasers" (demethylases, including ALKBH5 and FTO) are involved in the dynamic regulation process of m^6^A (Lan et al. 2019). The fat mass- and obesity-associated protein (FTO) is the first identified demethylation enzyme, involved in a variety of regulatory biological processes (Zhang et al. 2022). There is growing evidence that FTO promotes the development of a variety of cancers including acute myeloid leukemia (AML), melanoma, breast cancer and lung cancer (Li et al. 2017a, b; Castillo et al. 2012; Kaklamani et al. 2011; You et al. 2022). In addition, FTO is associated with self-renewal and immune evasion of cancer stem cells, and plays an essential role in the progression and metastasis of gastric cancer (Zhang et al. 2021). FTO is also involved in the proliferation, invasion and metastasis of gastric cancer cells, and has excellent prognostic value (Guan et al. 2020).m^6^A modifications are involved in the regulation of tumor stem cells (CSC) and tumor immune microenvironment leading to malignant phenotypes (Kumari et al. 2022). For example, in pancreatic cancer, FTO regulates the expression of CSC markers involved in pancreatic carcinogenesis (Garg et al. 2022).CD44, a gastric cancer stem cell marker, is not only involved in tumor growth and maintenance of cancer cell stemness, but also highly correlated with tumorigenicity, invasiveness, lymphatic metastasis and drug resistance of gastric cancer cells, which greatly affects the treatment and prognosis of gastric cancer (Wang et al. 2011; Qi et al. 2022).Interestingly, it was found that CD44 expression gradually increased during the process of Correa's cascade, and the abnormal alteration of CD44 was earlier than CD133, which is also a biomarker of gastric cancer stem cells, so CD44 is considered as a biomarker and key molecule of malignant transformation (Zavros 2017; Wang et al. 2011). However, the mechanism of CD44 involvement in malignant transformation of gastric mucosa is still unclear.

The biological significance of m^6^A in the malignant transformation of gastric epithelium due to chronic *H. pylori* infection and its potential regulatory mechanisms remain unclear. In this study, we investigated the role of FTO-mediated m^6^A modification in the development of malignant transformation of human normal gastric mucosal epithelial cells from chronic infection with Cag A^+^*H. pylori*.

## Methods

### Cell culture

Immortalized human gastric mucosal epithelial cells GES-1 (from the Cell Resource Center, Beijing, China) were used, and the cell culture media were supplemented with 10% fetal bovine serum (FBS, BI) and 1% penicillin–streptomycin (100 U/mL) with RPMI 1640 medium (Gibco), and the cells were cultured at 37 °C in a 5% CO_2_ thermostatic incubator.

### *Helicobacter pylori* culture

We used 2 types of previously collected *H. pylori* strains (Li et al. 2017a, b), one of which expresses Cag A protein and the other does not. All strains were cultured on Columbia blood agar plates containing sheep blood and *H. pylori* screening agent, and after 3–5 days of incubation at 37 °C in a microaerobic constant temperature environment, Cag A^−^*H. pylori* strains grew as tiny pinpoint-like dots (Supplementary Fig. S1A) and Cag A^+^*H. pylori* strains grew as thicker columnar colonies (Supplementary Fig. S1A). Colonies were scraped using an inoculation loop, DNA was extracted and identified by agarose electrophoresis (Supplementary Fig. S1B). After the bacterial growth was stabilized, the colonies were scraped into RPMI 1640 medium containing 10% FBS and used immediately.

### Co-culture of GES-1 with* H. pylori*

In the *H. pylori* chronic infection model (Yu et al. 2011; Liu et al. 2021; Lin et al. 2019), GES-1 cells were cultured in antibiotic-free cell culture medium and inoculated in 6-well plates (Corning) at a density of 3 × 10^5^ cells/well. At approximately 80% cell confluency, freshly collected *H. pylori* were suspended in antibiotic-free cell culture medium and added to the cells for co-culture. *H. pylori* and GES-1 were co-cultured at MOI (multiplicity of infection) = 1:1, 10:1, 100:1, respectively, and the original medium was replaced every 24 h with freshly collected *H. pylori*. Co-culture was continued for approximately 6 months in the chronic infection model. In the model of acute infection, *H. pylori* was co-cultured with GES-1 cells under the same conditions for 48 h. The bacterial concentration was calculated based on the optical density (OD) of 2 × 10^8^ colony forming units (CFU) at 660 nm. Cells were used for experimental analysis during the intervention period.

### Cell morphology observation

Co-cultured GES-1 was observed under an inverted phase contrast microscope, and morphological changes were observed in the normal, Cag A^−^*H. pylori* and Cag A^+^*H. pylori* chronically infected cell.

### CCK-8 assay for cell viability and proliferation

Cell viability and proliferation were assessed using the CCK-8 kit (Biosharp). Cells were inoculated into 96-well plates (100 µl/well) at a density of 3 × 10^4^ cells/ml and incubated at 0 h, 12 h, 48 h and 72 h with 10µl of CCK-8 solution in an incubator containing 5% CO_2_ at 37 °C for 2 h. The absorbance was measured at 450 nm using a multifunctional enzyme marker. OD values were positively correlated with the number of cell proliferation and subsequently plotted cell growth curve.

### Cell colony formation assay

Cells were turned into individual cells using trypsin (Biosharp), counted and then inoculated at 300–500 cells/well in 6-well plates, grown for 15 days by adding complete medium, fixed in 4% paraformaldehyde (Biosharp) for at least 30 min, stained with crystal violet (GCLONE) and then counted for analysis.

### Transwell assay

Cell migration was determined by transwell assay. 100 µl of cell suspension was added to each well of the apical chamber. At the same time, 500 µl of conditioned medium containing 30% FBS was added to the basolateral chamber. After incubation at 37 °C for 48 h, transwell plates were fixed with 4% paraformaldehyde for 30 min, followed by staining the cells with crystal violet for 30 min at room temperature. The cells were wiped off the surface with a cotton ball. Finally, cells were microscopically observed and counted using at least four randomly selected fields of view.

### EdU cell proliferation assay

Cell proliferation capacity was assayed according to the steps in the instructions (Meilunbio). An equal volume of EdU reagent (2X, 50 µM) in complete medium configuration was added to the cell culture medium, incubated for 2 h at 37 °C and then fixed using paraformaldehyde, 0.5% Triton-100 permeabilized cells and stained using 488-Azide with Hoechst 33,342.

### Live and dead cell double staining

The cells were washed thoroughly with 1X Assay, and the staining solution was prepared according to the Calcein AM/PI Double Stain Kit (MKBio), added to the cell suspension and incubated at 37 °C for 15 min. The cells were double stained and labeled with Calcein-AM and Propidium Iodide (PI) for live and dead cells, respectively, for analysis at the live and dead cell levels.

### Western blot

Proteins were obtained using RIPA lysis buffer with 1 mM PMSF and protein concentration was determined by BCA protein quantification method. Proteins were separated by SDS–PAGE electrophoresis and transferred to PVDF membranes. The membranes were incubated with 5% skim milk for 1 h at 4 °C and overnight with the following primary antibody. Then, the membranes were incubated with horseradish peroxidase-conjugated anti-rabbit IgG or anti-mouse IgG for 1 h. Protein bands are detected using the ECL kit (Biosharp).

The primary antibodies and secondary antibodies used were as follows: FTO (ZENBIO), CD44 (ZENBIO) and horseradish peroxidase-conjugated anti-IgG secondary antibodies (Cell Signalling Technology).

### Quantitative real-time PCR (qRT-PCR)

Total RNA was extracted and cDNA was obtained according to the reverse transcription instructions (Vazyme). Primer sequences were as follows: GAPDH, 5′-GGTCACCAGGGCTGCTTTA-3′ (Forward); 5′-GGATCTCGCTCCTGGAAGATG-3′(Reverse); FTO,5′-ACTTGGCTCCCTTATCTGACC-3′(Forward); 5′-TGTGCAGTGTGAGAAAGGCTT-3′(Reverse); CD44, 5′-CTGCCGCTTTGCAGGTGTA-3′ (Forward); 5′-CATTGTGGGCAAGGTGCTATT-3′ (Reverse); and the relative expression of target genes at the mRNA level was calculated by the 2-ΔΔCT method, using GAPDH as an endogenous control.

### Immunohistochemistry

Specimens of gastric cancer from primary gastric cancer patients who underwent surgery at The Third Xiangya Hospital were collected, and the specimens were taken at the tumor and > 15 cm from the tumor margin, respectively, and the tissues were fixed in paraformaldehyde and paraffin-embedded sections were used for immunohistochemical diagnosis. This study was approved by the ethics committee of The Third Xiangya Hospital to be conducted.

### Flow cytometry analysis

Cells were digested using EDTA-free trypsin, washed twice with Phosphate Buffered Saline (PBS), resuspended by adding 500 µl of Binding Buffer, incubated together with 5 µl of Annexin V and 5 µl of PI (KeyGEN BioTECH), for 15 min at room temperature, and analyzed for apoptosis by flow cytometry.

### In vivo tumor xenograft study

For the tumor growth analysis, GES-1 cells were subcutaneously injected into BALB/c nude mice (male, 5 weeks), and tumor volumes were monitored every 5 days. Tumor volumes were estimated based on length and width and calculated using the following formula: tumor volume = (length × width^2^)/2. About 1 month later, the nude mice were sacrificed, and then tumors were excised, pictured, and weighed.

### m^6^A RNA dot blot assay

The mRNA samples are dissolved in nuclease-free water, denatured at 65 °C for 5 min, then, transferred to Nylon Transfer Membrane and crosslinked by UV, incubated with 5% skim milk for 1 h, then incubated overnight at 4 °C with an antibody specific for m^6^A and finally incubated with HRP-linked anti-rabbit IgG or anti-mouse IgG for 1 h. Staining with approximately 0.2% methylene blue was used as a control.

### MeRIP–qPCR

After obtaining high quality total cellular RNA, Methylated RNA Immunoprecipitation Assay (MeRIP) were performed. m^6^A RNA fragments were enriched and purified using the EpiQuik™ CUT&RUN m^6^A RNA Enrichment kit (Epigentek), and CD44 expression at the RNA level was detected by PCR.

### Statistical analysis

Data were expressed as the mean ± SEM from at least three independent experiments. Student's t-test was used for two-group comparisons. For multiple comparisons, Two-way analysis of variance (ANOVA) was performed using GraphPad Prism^®^ version 8.0 software. *p* values < 0.05 were considered statistically significant.

## Results

### Increased apoptosis of GES-1 after acute infection with *H. pylori*

**Fig. 1:**
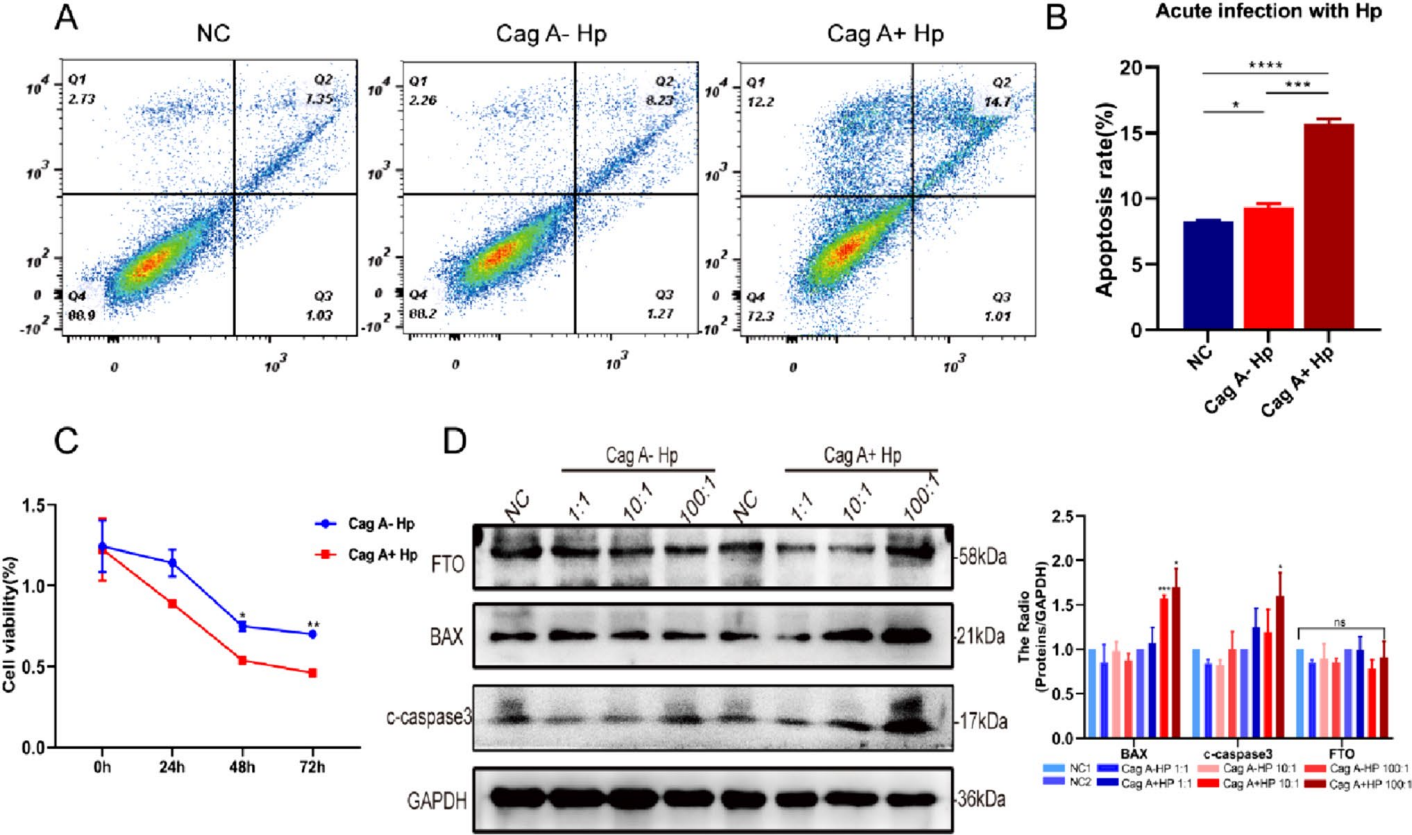
Increased apoptosis in acute *H. pylori* infected GES-1 cells.** A** Flow cytometry results of GES-1 cells after 48 h of acute *H. pylori* infection, **B** statistical analysis. **C** CCK-8 assay for GES-1 cell viability. **C** BAX, c-caspase3 and FTO were determined by Western blotting after acute infection with *H. pylori* in GES-1 cells. Hp: *H. pylori*, *p < 0.05, **p < 0.01, ***p < 0.001

*H. pylori* relies on a powerful chemotactic motility system to colonize the host gastric mucosa, releasing a variety of virulence factors that resist host immune clearance, induce inflammatory responses and damage host mucosal tissue. Flow cytometry showed a significant increase in GES-1 apoptosis within 48 h of *H. pylori* infection, which was more pronounced in Cag A^+^*H. pylori* infection (Fig. 1A, B). The CCK-8 assay revealed a greater effect of Cag A^+^*H. pylori* on GES-1 cell activity at 48 h and 72 h of *H. pylori* infection (Fig. 1C). Further Western Blot results showed that the expression of Bax and Cleaved Caspase-3 increased in GES-1 cells with increasing concentrations of Cag A^+^*H. pylori* acute infection compared to control and Cag A^−^*H. pylori* infected groups (Fig. 1D). These tend to suggest damage to the host gastric mucosa under the acute impact of *H. pylori* infection.

### Chronic Cag A^+^*H. pylori* infection induces malignant transformation of GES-1

**Fig. 2:**
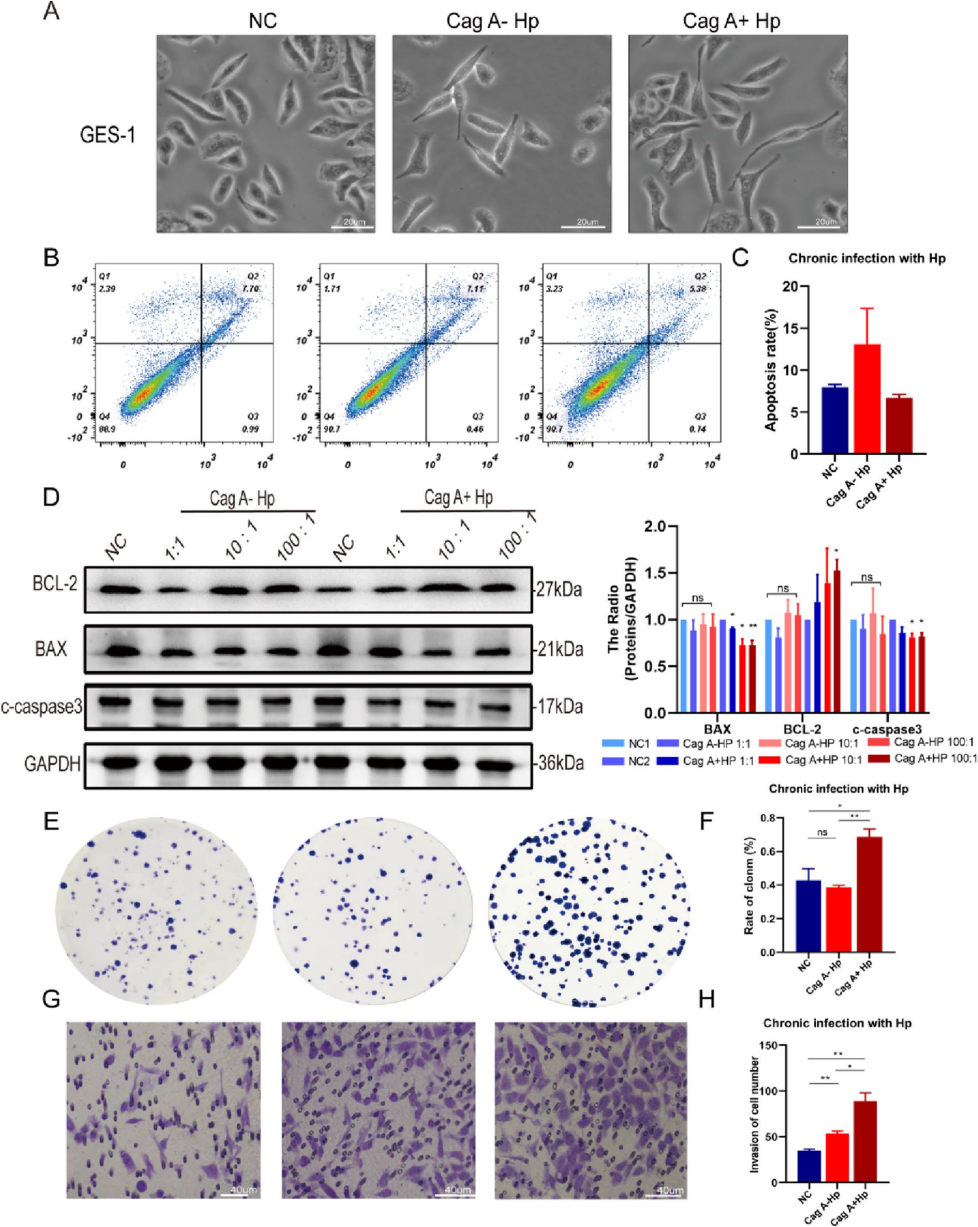
Chronic Cag A^+^*H. pylori* infection induces malignant transformation of GES-1.** A** GES-1 cells show a hummingbird phenotype after chronic infection with Cag A^+^*H. pylori*. **B** Reduced apoptosis in GES-1 cells detected by flow cytometry after chronic infection with Cag A^+^*H. pylori*. **C** Statistical analysis. **D** Detection of BCL-2, BAX and c-caspase3 protein levels in GES-1 cells after chronic infection with *H. pylori* by Western blot. **E, G** Enhanced proliferation and migration of GES-1 cells in Cag A^+^*H. pylori* chronic infection detected by colony formation and Transwell assay. **F, H** Statistical analysis. ns: no statistical significance, *p < 0.05, **p < 0.01

Normal GES-1 shows shuttle-shaped or polygonal cells with regular shape and clear edges. GES-1 cells are bipolarly prolonged and cellularly narrowed after chronic *H. pylori* infection. Morphological changes were more pronounced in GES-1 cells chronically infected with Cag A^+^*H. pylori*, with linearly prolonged cell poles and hummingbird-like cells (Fig. 2A and Supplementary Fig. S1C). It has been shown that *H. pylori* synthesizes Cag A and binds to SHP-2 via T4SS transport into host cells, disrupting tight intercellular junctions, rearranging the cytoskeleton, and altering cell morphology. In addition, the cells with hummingbird morphology tend to have more potential for transformation.

**Fig. 3:**
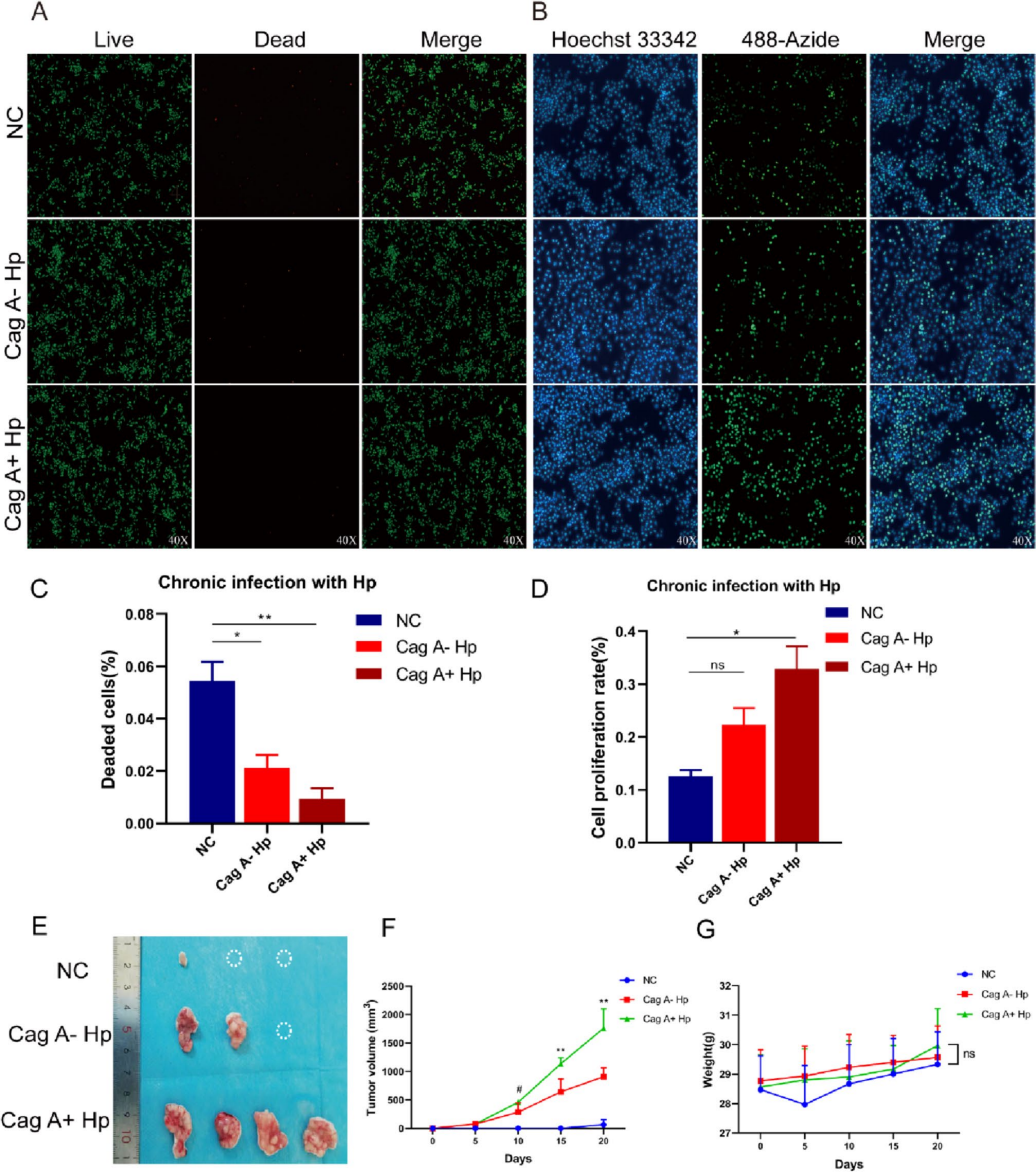
Chronic infection with Cag A^+^*H. pylori* induces malignant transformation of GES-1. **A** Live-dead cell staining reveals reduced GES-1 cell death after chronic infection with Cag A^+^*H. pylori*. **B** EdU Staining finds increased proliferation of GES-1 cells after chronic infection with Cag-A^+^*H. pylori*. **C, D** Statistical analysis. **E, F** Chronic Cag A^+^*H. pylori* infection induces the tumorigenicity of GES-1 cells in vivo, body weight, tumor volume and anatomical tumor images of nude mice were recorded and analyzed. ns: no statistical significance, #: NC and Cag A^+^*H. pylori* groups were statistically significant. *p < 0.05, **p < 0.01

Acute Cag A^+^*H. pylori* infection affects cell viability and contributes to apoptosis of GES-1 cells. However, after chronic infection with Cag A^+^*H. pylori*, flow cytometry revealed reduced GES-1 apoptosis (Fig. 2B, C) and decreased expression of apoptotic proteins (Fig. 2D), suggesting a disruption of the programmed cell death process in response to long-term *H. pylori* infection stimulation. In addition, sustained Cag A^+^*H. pylori* stimulation caused malignant transformation of normal GES-1 cells, such as increased abnormal proliferation, invasiveness and tumorigenicity. GES-1 cells in the Cag A^+^*H. pylori* chronic infection group showed stronger cell proliferation and invasive ability in colony formation and transwell assays (Fig. 2E–H). In addition, EdU Staining revealed increased proliferation and enhanced DNA replication activity of GES-1 cells under chronic stimulation of Cag A^+^*H. pylori* (Fig. 3B, D). Live and dead cell staining suggested that the number of cell death in the Cag A^+^*H. pylori* chronic infection group was less than that in the Cag A^−^*H. pylori* chronic infection group and the control group (Fig. 3A, C). In a subcutaneous tumorigenic assay in nude mice, Cag A^+^*H. pylori* chronically stimulated GES-1 cells exhibited tumorigenicity similar to tumor cells (Fig. 3E–G).

### FTO is involved in the malignant transformation of GES-1 under persistently stimulation of Cag A^+^*H. pylori*

**Fig. 4:**
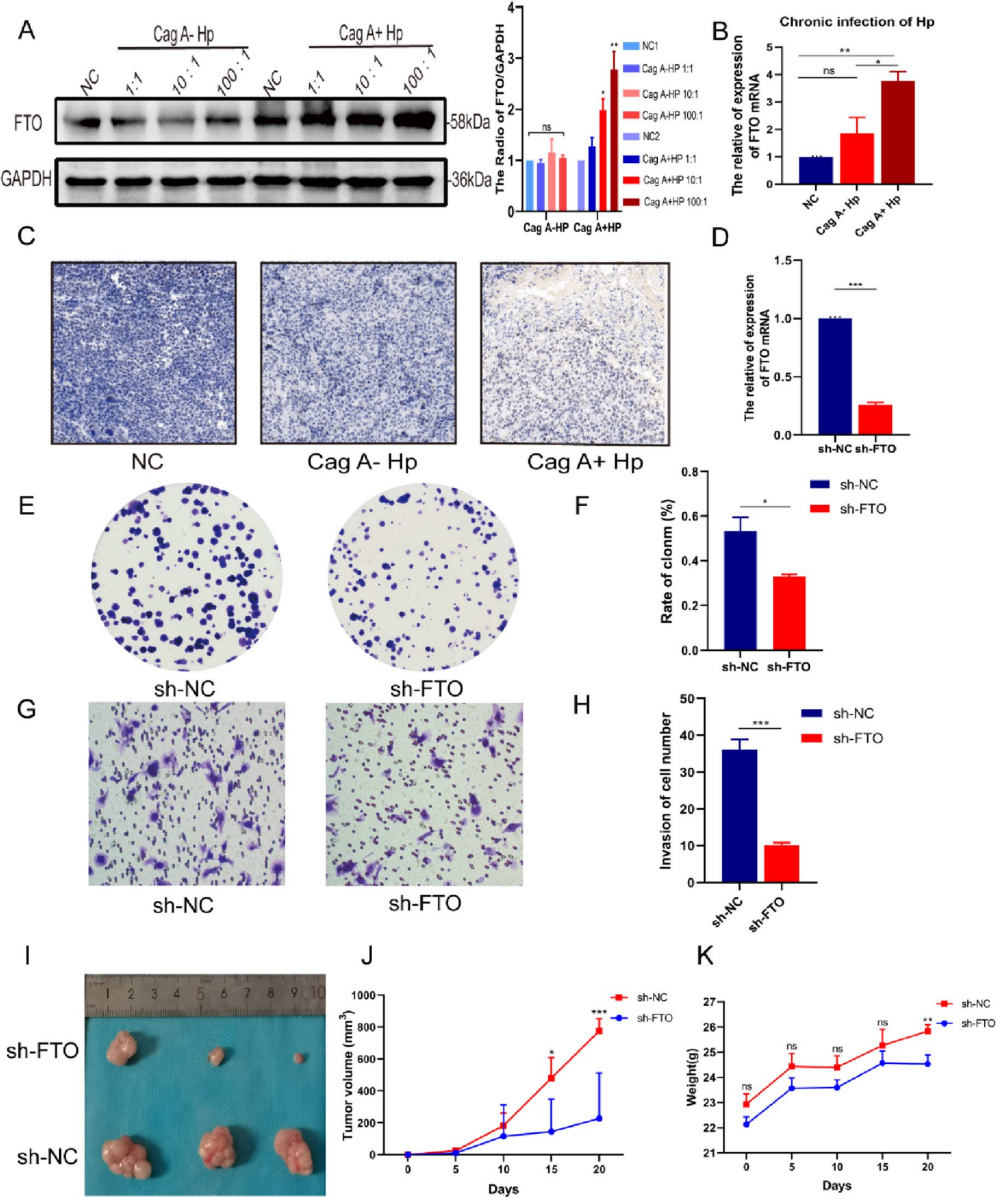
FTO is involved in the malignant transformation of GES-1 in response to sustained stimulation by Cag A^+^*H. pylori*. **A, B** Elevated FTO protein and RNA expression after chronic Cag A^+^*H. pylori* Infection. **C** IHC staining of tumor tissues from nude mice verified that the FTO expression was elevated after chronic infection with Cag A^+^*H. pylori*. **D** Efficiency of knockdown of FTO in Cag A^+^*H. pylori* chronically infected GES-1 cells by qRT-PCR. **E, G** Knockdown of FTO inhibited cell proliferation and migration of GES-1 cells measured by colony formation and Transwell assay. **F, H** Statistical analysis. **I, J** knockdown of FTO inhibited the tumorigenicity of GES-1 cells in nude mice. ns: no statistical significance, *p < 0.05, **p < 0.01, ***p < 0.001

With increasing duration of infection, the expression of FTO was significantly higher in Cag A^+^*H. pylori* infection group in comparison with the control and Cag A^−^*H. pylori* infection groups, and showed a dose-dependent relationship with the concentration of Cag A^+^*H. pylori* infection (Fig. 4A) and higher expression at the RNA level (Fig. 4B). GES-1 was more tumorigenic in vivo after chronic infection with Cag A^+^*H. pylori*, and immunohistochemistry assays in mouse tumors suggested higher FTO expression in the Cag A^+^*H. pylori* chronic infection group (Fig. 4C). After we down-regulated FTO expression in the Cag A^+^*H. pylori* chronic infection group using lentiviral transfection and confirmed FTO low expression by qRT-PCR (Fig. 4D), we found that inhibition of FTO expression rescued the signs of malignant transformation of GES-1 cells caused by Cag A^+^*H. pylori* chronic infection with abnormal proliferation, cell migration ability and tumorigenicity in animal assays. Functionally, silencing FTO inhibits cell proliferation and metastasis. Reduced cell proliferation was observed by colony formation assays (Fig. 4E, F), and transwell assays revealed a significant inhibition of cell metastatic ability (Fig. 4G, H), and the effect of FTO on the tumorigenicity of GSE-1 cells chronically infected with Cag A^+^*H. pylori* was also verified in in vivo experiments in animals. In tumor xenograft studies, we found that normal GES-1 hardly formed tumors, while GES-1 cells in the Cag A^+^*H. pylori* chronic infection group could form significant subcutaneous tumors and were statistically significant with the Cag A^−^*H. pylori* chronic infection group (Fig. 4I–K). These results suggest that chronic infection with Cag A^+^*H. pylori* induces morphological changes in GES-1 cells and significantly increases the proliferation and invasive capacity of GES-1 cells, which are more tumorigenic in nature, and that FTO is involved in the malignant transformation process of GES-1.

### FTO is involved in CD44 mRNA m^6^A modification in Cag A^+^*H. pylori* chronic infection

**Fig. 5:**
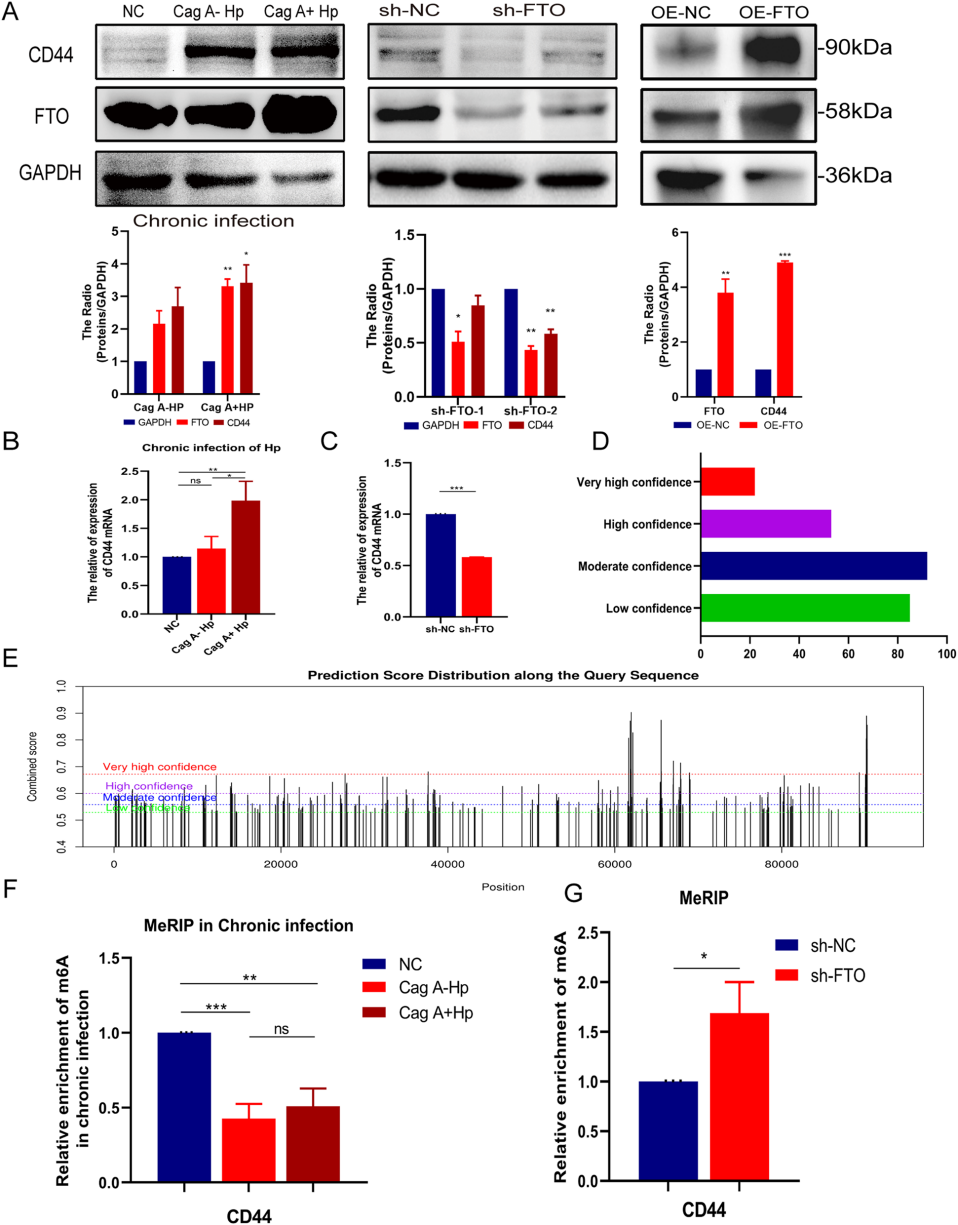
FTO is involved in CD44 mRNA m^6^A modification in Cag A^+^*H. pylori* chronic infection.** A** Western blot showed that CD44 expression increased with FTO after chronic infection of GES-1 cells by Cag A^+^*H. pylori*, and that overexpression or knockdown of FTO promoted or suppressed the protein expression level of CD44. **B, C** Increased CD44 RNA levels after chronic infection with Cag A^+^*H. pylori* and decreased CD44 RNA expression after knockdown of FTO. **D, E** m^6^A modification site predictor SRAMP was used to predict the m^6^A modification site in the CDS region of CD44. **F, G** Enrichment and validation of m^6^A-methylated RNA fragments by MeRIP–qPCR in GES-1 cells after chronic infection with *H. pylori*. The level of CD44 m^6^A modification decreased when FTO was highly expressed and increased after FTO knock-out, suggesting a role for m^6^A modification of CD44 by FTO. ns: no statistical significance, *p < 0.05, **p < 0.01, ***p < 0.001

GES-1 cells chronically infected by Cag A^+^*H. pylori* showed a significant increase in CD44 expression at the RNA and protein levels (Fig. 5A, B). CD44 is a recognized tumor stem cell marker molecule, and its increased expression predicts a malignant transformation change of GES-1 cells under continuous stimulation of Cag A^+^*H. pylori* (Ryu et al. 2012). CD44 is not only a marker of gastric cancer CSC, it is functionally participating in the tumorigenicity of gastric cancer cells (Takaishi et al. 2009). A number of studies have found that CD44 is progressively elevated in the Correa's cascade model and plays an active role in the development of gastric cancer (He et al. 2022; Yang et al. 2013; Choi et al. 2019). Daniel Brungs et al. found that CD44 ^+^ gastric cancer cells showed enhanced resistance to chemotherapy or radiation-induced cell death and were associated with poor prognosis (Brungs et al. 2016). Meanwhile, we found that CD44 expression was significantly correlated with FTO expression. When we silenced the FTO expression of GES-1 after chronic infection of *H. pylori*, the expression of CD44 was suppressed, and the expression level of CD44 was significantly increased after exogenous supplementation of FTO (Fig. 5A). qRT-PCR at the RNA level revealed a positive correlation between CD44 expression levels and FTO expression, and silencing of FTO hindered CD44 expression levels in Cag A^+^*H. pylori* chronically infected GES-1 cells (Fig. 5B, C).

To further explore the role of FTO-mediated m^6^A methylation in Cag A^+^*H. pylori* chronically infected GES-1 cells, we predicted the m^6^A binding site of CD44 by SRAMP website and found an abundant m^6^A binding site at the 3' UTR of CD44 (Fig. 5D, E). We enriched m6A-RNA fragments in GES-1 cells by methylated RNA Immunoprecipitation assay (MeRIP), and MeRIP–qPCR revealed that FTO mediated CD44 mRNA m^6^A modification. The results showed that chronic infection of Cag A^+^*H. pylori* induced high expression of FTO, and m^6^A modification of CD44 was reduced when FTO was up-regulated, but significantly increased after FTO expression was silenced (Fig. 5F, G). The above results suggest the enrichment of m^6^A RNA in GES-1 after chronic infection with Cag A^+^*H. pylori*, and verified the interaction between FTO and CD44 mRNA, suggesting that FTO, as a demethylating enzyme, regulates CD44 mRNA in a m^6^A-dependent manner to involve in malignant transformation of gastric mucosal epithelial cells.

### FTO is enriched in gastric cancer and associated with poor prognosis

**Fig. 6:**
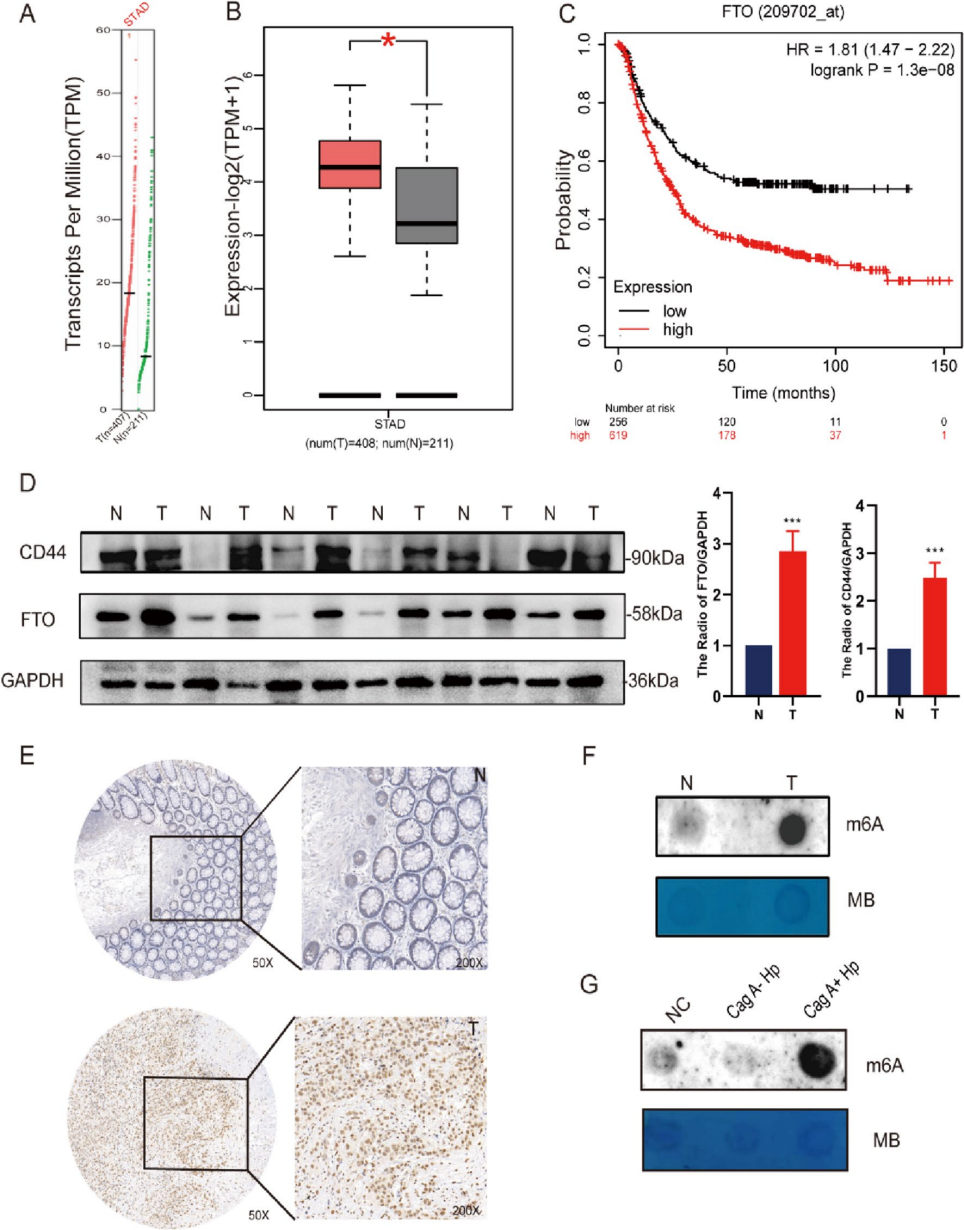
FTO expression is up-regulated in GC tissues and its prognostic value in GC.** A, B** Expression of FTO in gastric cancer tissues and **C** prognostic value of FTO in gastric cancer tissues were analyzed in the clinical database GEPIA. **D** Expression of FTO and CD44 protein levels in GC tissues and paracancerous tissues by Western blotting. **E** Analysis of FTO protein level expression in GC tissues and paraneoplastic tissues by immunohistochemistry. **F, G** Total m^6^A levels in gastric cancer tissues and subcutaneous tumor tissues in nude mice were analyzed by m^6^A RNA Dot Blot Assay, and MB (methylene blue) staining was used as the loading control

The outcome of malignant transformation of normal gastric epithelial cells often points to the development of gastric cancer. Finally, we argue a strong link between FTO and gastric cancer, further suggesting the involvement of FTO in the malignant transformation of normal gastric epithelium and even in the development of tumorigenesis. We used the GEPIA database to analyze and found that FTO expression was significantly elevated in GC tissues (Fig. 6A, B), and then we analyzed the Kaplan–Meier Plotter database to correlate FTO expression levels with overall survival (OS) of GC patients and found that high FTO expression tended to predict poor survival prediction (log-rank *p* < 0.001) (Fig. 6C). In gastric cancer tissues, Western blot revealed that the expression level of FTO and CD44 was significantly higher than that of adjacent tissues (Fig. 6D), and in immunohistochemistry, FTO expression was significantly elevated in GC patients, with positive staining mainly concentrated in GC cell nuclei (Fig. 6E). Total m^6^A levels were elevated in gastric cancer tissues and in subcutaneous tumor tissues of mice in Cag A^+^*H. pylori* chronic infection group (Fig. 6F, G).

## Discussion

GC is the fifth most common malignancy in the world, characterized by high mortality, poor differentiation and poor prognosis (Digklia and Wagner 2016; Rugge et al. 2019). Chronic *H. pylori* infection is the main cause of gastric cancer (Correa 1992; Choi et al. 2018), but the process from *H. pylori* chronic infection-induced malignant transformation of gastric mucosa, precancerous lesions to gastric carcinogenesis is not clear.

**Fig. 7:**
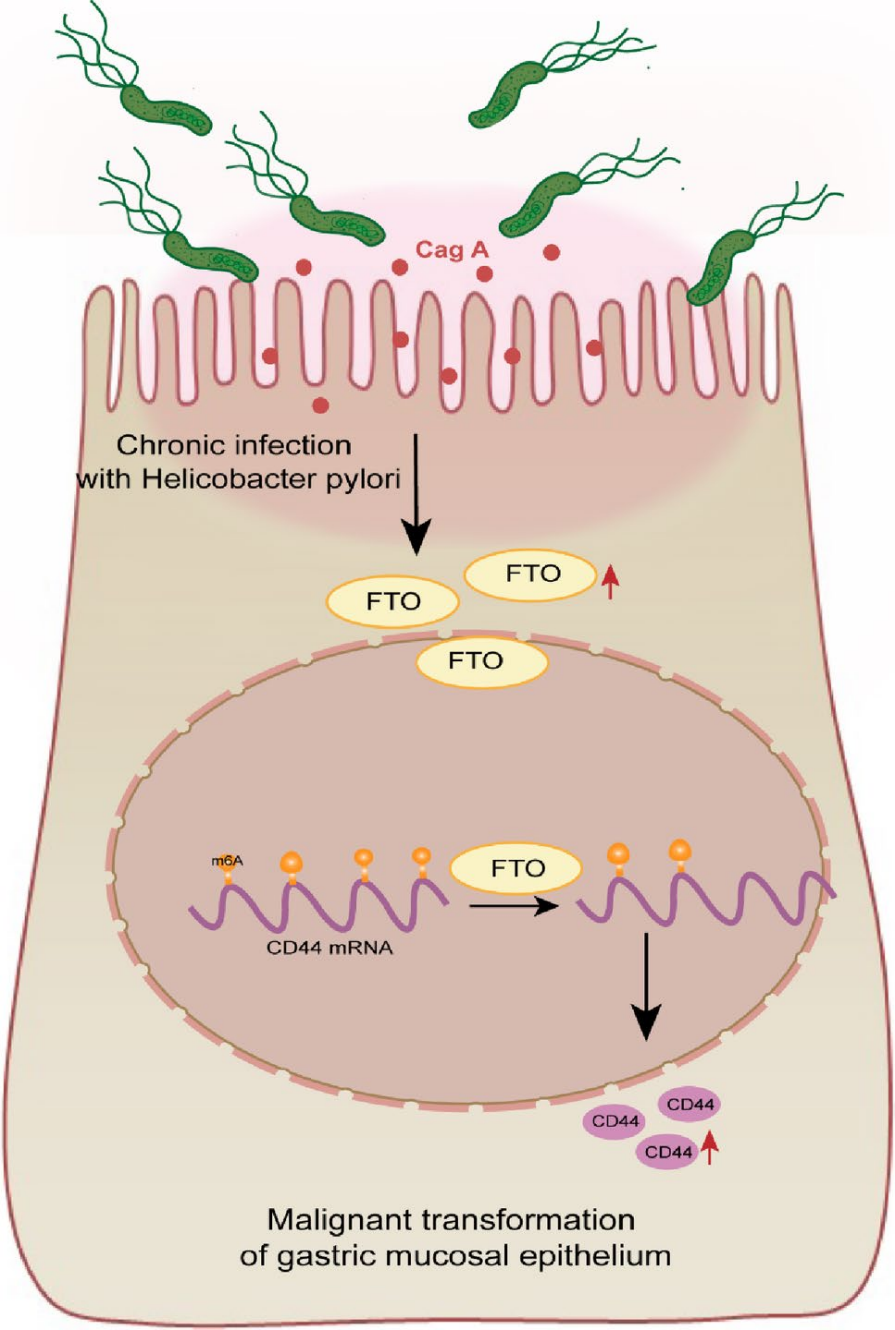
Regulatory axis of Cag A^+^*H. pylori*/FTO/CD44 is involved in the malignant transformation of GES-1

Gastric carcinogenesis and progression are also affected by epigenetic modifications. FTO is an independent prognostic marker for gastric cancer and also the most potent prognostic risk factor among all m^6^A regulators (Wang et al. 2021; Zhao et al. 2021). GC patients with high expression of FTO were often accompanied by poor TNM staging and prognosis (Su et al. 2019). In addition, FTO can dynamically regulate the structure and function of RNA by reducing m^6^A modifications, increasing the translational expression of oncogenes or triggering transcriptional changes in oncogenes to induce cellular deterioration and promote the development of malignancy (Zou et al. 2019). Yang Z et al. found that FTO promotes the stability of MYC mRNA by decreasing the m^6^A methylation of MYC in GC cells, thereby accelerating the proliferation, migration and invasive properties (Yang et al. 2021). FTO was found to promote GC metastasis by regulating m^6^A levels of integrin β1 (ITGB1) to upregulate ITGB1 expression (Wang et al. 2021). Similarly, our study found that m^6^A levels were altered in both malignantly transformed gastric epithelial cells and gastric cancer tissues, and CD44 was positively correlated with the expression level of FTO. In addition, combined with the prediction of m^6^A modification sites, we identified CD44 as a possible demethylation target of FTO. This result supports that FTO plays an important tumor-promoting role before gastric carcinogenesis, which has important implications for the diagnosis and treatment of gastric cancer (Fig. 7).

In this study, we found that in Cag A^+^*H. pylori* chronically stimulated GES-1 cells, upregulated FTO promoted CD44 expression by regulating m^6^A modification of CD44 RNA, accompanied by abnormal changes in cell morphology and enhanced proliferation invasion, which were associated with malignant transformation of normal gastric epithelium. CD44, a member of the CAM family, is a major receptor for hyaluronic acid (HA) and is normally involved in cell signaling-mediated cell proliferation, invasion and migration (Senbanjo and Chellaiah 2017). CD44 is also a marker molecule for many tumor–stem cells, including gastric cancer. Low differentiation of tumor and poor prognosis of gastric cancer correlate with CD44. CD44 expression rate was found to be as high as 80% in primary gastric cancer resection specimens, and gastric cancer with high CD44 expression was associated with higher level of clinicopathological features and worse prognosis (Brungs et al. 2016). Shigeo Takaishi et al. found that CD44^+^ gastric cancer cells exhibited self-renewal properties and differentiation of stem cells. In vivo, CD44^+^ GC cells injected dermally and intragastrically were more tumorigenic in mice, and the same was verified in a xenograft test in mice (Takaishi et al. 2009).

## Conclusion

Overall, our findings suggest the involvement of Cag A^+^*H. pylori*/FTO/CD44 in the malignant transformation of GES-1 and reveal the possibility of FTO in promoting gastric carcinogenesis by modifying CD44 mRNA methylation, and FTO may be a predictor and therapeutic target for malignant transformation of gastric epithelium, providing a new perspective for the prevention and treatment of gastric cancer. However, the specific mechanisms regulating the process of malignant transformation of the gastric mucosa still need further investigation.

## Supplementary Information

Below is the link to the electronic supplementary material.

Supplementary file1 (DOCX 242 kb)

## Data Availability

The data sets generated during and/or analysed during the current study are available from the corresponding author on reasonable request.
