# Supplementary material for: FTO-mediated m6A modification promotes malignant transformation of gastric mucosal epithelial cells in chronic Cag A+ Helicobacter pylori infection
Source: J Cancer Res Clin Oncol. 2023 Mar 15;149(10):7327–40. doi: 10.1007/s00432-023-04684-4 (PMC10374804; doi:10.1007/s00432-023-04684-4)
Supplement: Supplementary file 1 — Supplementary file1 (DOCX 242 kb) [file 432_2023_4684_MOESM1_ESM.docx]

**
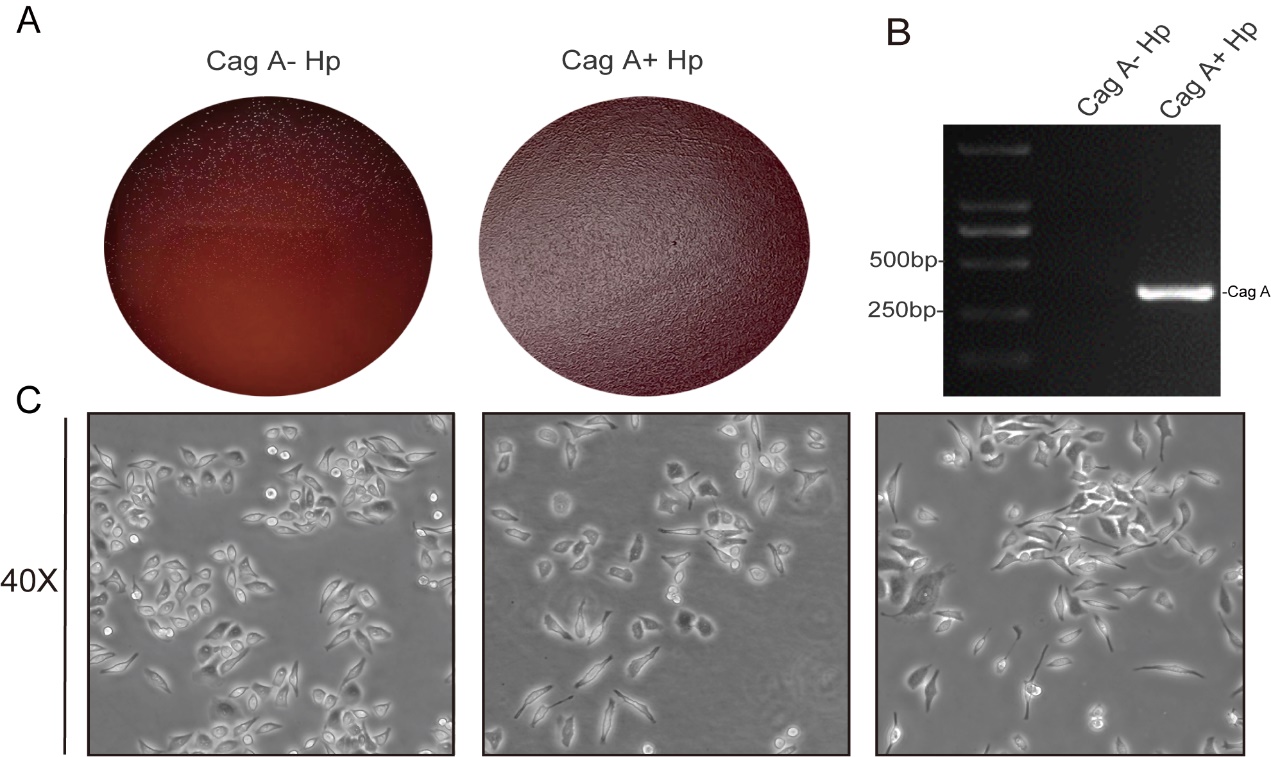
**

**Fig S1 Morphological observation.** **A** Morphological observation of Cag A^-^*H. pylori* and Cag A^+^*H. pylori* in Petri dishes, **B** Identification of Cag A^-^*H. pylori* and Cag A^+^*H. pylori*. **C** From left to right, the morphological changes of GES-1 after chronic infection with Cag A^-^*H. pylori* and Cag A^+^*H. pylori* in control group (observed under microscope at 40x).
